# Initial experience with a SiPM-based PET/CT scanner: influence of acquisition time on image quality

**DOI:** 10.1186/s40658-018-0207-x

**Published:** 2018-04-18

**Authors:** Ida Sonni, Lucia Baratto, Sonya Park, Negin Hatami, Shyam Srinivas, Guido Davidzon, Sanjiv Sam Gambhir, Andrei Iagaru

**Affiliations:** 1 Division of Nuclear Medicine and Molecular Imaging, Department of Radiology, Stanford University, 300 Pasteur Drive, Stanford, CA 94305 USA; 2 Molecular Imaging Program at Stanford, Department of Radiology, Stanford University, Stanford, CA USA; 3 Molecular Biophysics and Integrated Bioimaging, Lawrence Berkeley National Lab, Berkeley, CA USA

**Keywords:** PET/CT, Acquisition time, Silicon photomultipliers, Detectors

## Abstract

**Background:**

A newly introduced PET/CT scanner (Discovery Meaningful Insights—DMI, GE Healthcare) includes the silicon photomultiplier (SiPM) with time-of-flight (TOF) technology first used in the GE SIGNA PET/MRI. In this study, we investigated the impact of various acquisition times on image quality using this SiPM-based PET/CT.

**Methods:**

We reviewed data from 58 participants with cancer who were scanned using the DMI PET/CT scanner. The administered dosages ranged 295.3–429.9 MBq (mean ± SD 356.3 ± 37.4) and imaging started at 71–142 min (mean ± SD 101.41 ± 17.52) after administration of the radiopharmaceutical. The patients’ BMI ranged 19.79–46.16 (mean ± SD 26.55 ± 5.53). We retrospectively reconstructed the raw TOF data at 30, 60, 90, and 120 s/bed and at the standard image acquisition time per clinical protocol (180 or 210 s/bed depending on BMI). Each reconstruction was reviewed blindly by two nuclear medicine physicians and scored 1–5 (1—poor, 5—excellent quality). The liver signal-to-noise ratio (SNR) was used as a quantitative measure of image quality.

**Results:**

The average scores ± SD of the readers were 2.61 ± 0.83, 3.70 ± 0.92, 4.36 ± 0.82, 4.82 ± 0.39, and 4.91 ± 0.91 for the 30, 60, 90, and 120 s/bed and at standard acquisition time, respectively. Inter-reader agreement on image quality assessment was good, with a weighted kappa of 0.80 (95% CI 0.72–0.81). In the evaluation of the effects of time per bed acquisition on semi-quantitative measurements, we found that the only time point significantly different from the standard time were 30 and 60 s (both with *P* < 0.001). The effects of dose and BMI were not statistically significant (*P* = 0.195 and 0.098, respectively). There was a significant positive effect of time on SNR (*P* < 0.001), as well as a significant negative effect of weight (*P* < 0.001).

**Conclusions:**

Our results suggest that despite significant delays from injection to imaging (due to comparison with standard PET/CT) compared to standard clinical operations and even in a population with average BMI > 25, images can be acquired as fast as 90 s/bed using the SiPM PET/CT and still result in very good image quality (average score > 4).

## Background

Positron emission tomography (PET) is a functional imaging technique developed in the 1970s that is now an indispensable tool in the evaluation of biological functions at molecular level [1, 2]. PET plays a particularly crucial role in cancer imaging, for diagnostic, staging, and prognostic purposes [3]. After initial use as PET alone, the first hybrid PET/CT scanner was introduced and evaluated clinically in 2000 [4]. Hybrid PET/CT scanners benefit from the addition of structural information derived from CT and its important ability to provide maps of tissue attenuation for correction of the PET images, which have significantly improved the duration of the exam, patients’ compliance, and ultimately diagnostic accuracy [5]. A substantial improvement in PET/CT is time-of-flight (TOF) capability. TOF allows for better localization of the annihilation event along the lines-of-response due to the use of fast detectors and ultimately improves the tomographic reconstruction and image quality [6]. More recently, simultaneous PET/MRI scanners have also been developed with the expectation to improve workflows and clinical usefulness in those fields where the properties of MRI are valuable [7–10]. Such combined and simultaneous scanners required remarkable modifications in PET technology over the years to move away from photomultiplier tubes.

Since their initial development to current times, most PET scanners were built with scintillation crystals coupled with PMTs. However, in recent years SiPM detectors emerged as a new technology foreseen as a promising alternative to PMTs [11]. These photodetectors offer several advantages including relative indifference to the magnetic fields of MR which made them attractive for the use in hybrid PET/MR systems, compact size, and potentially low production costs [11, 12]. Furthermore, SiPM are characterized by excellent intrinsic time resolution and high photon-detection efficiency, which made them the most favorable devices to be coupled with TOF reconstruction [12]. The differences between standard and SiPM PET detectors are significant. SiPMs having a small physical profile and a negligible electronic noise, offering high gain, and requiring low operating voltage offer several advantages over previously used technologies such as photomultiplier tubes (PMTs) and avalanche photodiodes (APDs) [13, 14]. Among the potential advantages of SiPM-based PET/CT over standard PET/CT are decreased required dosage of PET radiopharmaceuticals, higher sensitivity, and temporal resolution. Our group has previously reported the first experiences using SiPM-based detectors in the SIGNA PET/MRI [15, 16].

We installed in August 2016 the first SiPM-based clinical PET/CT (Discovery Meaningful Insights (DMI), GE Healthcare, Waukesha, WI, USA) that brings together SiPM and TOF [17]. Our hypothesis was that one can scan faster using the DMI without a compromise in image quality. Here, we report our initial results from the evaluation of the impact of various acquisition times on image quality using this SiPM-based PET/CT scanner.

## Methods

### Patient population

The study was approved by the Institutional Review Board (IRB) at Stanford University. Written informed consent was obtained from each participant before enrollment. Between September and December 2016, adult patients (18 years of age or older) referred to the Division of Nuclear Medicine and Molecular Imaging at Stanford University for a clinical oncological ^18^F-fluorodeoxyglucose (^18^F-FDG) PET/CT examination were selected and enrolled in our study. Patients unable to lie still for the duration of the exam and pregnant women were excluded.

### Study design and PET/CT protocol

This is a retrospective analysis of data acquired prospectively in a study comparing the performance of standard PET/CT scanners (GE Discovery 600 PET/CT scanner and GE Discovery 690 PET/CT scanner) and the GE DMI SiPM-based PET/CT scanner. Patients underwent a single injection of ^18^F-FDG, followed by a dual-imaging PET protocol, including first the standard PET/CT, and immediately after the SiPM-based PET/CT. The DMI did not have FDA-clearance at the time the protocol was approved by the local ethics committee; therefore, standard PET/CT was always used first at standard time for oncological imaging.

Here, we only report on the PET datasets from the DMI PET/CT scanner. These were acquired in list mode. Subsequently, the PET list mode data was reprocessed to produce the following sets of sinograms: 30, 60, 90, and 120 s per bed (sec/bed). The reference standard acquisition time (reference time) varied between 180 and 210 s per bed, depending on the patients’ BMI, as recommended by the manufacturer and to match the local clinical practice used for the D600 and D690 PET/CT scanners. All data sets were reconstructed using TOF with point spread function (PSF) and 3 iterations, 16 subsets, and a 5-mm post-filter, standard normalized *z* axis filtering, in a 256 × 256 matrix (voxel dimensions of 2.73 × 2.73 × 2.79). A 17-slice overlap was used on the DMI PET/CT scanner, which translates to 24% percentage of bed overlap. The average number of beds per patient, calculated dividing patient’s height by axial field of view (20 cm) for a head-to-toe scan, was 9, whereas for head-to-mid-thigh acquisitions, it was 6. The CT protocol utilized a 40-mm detector coverage, with a slice thickness of 3.75 mm, slice interval of 2.79 mm to match the PET slice thickness, and a pitch of 0.984 with a 0.5 s rotation time. The kV was set to 120 and the mA was set to modulate between 100 and 165 with a Noise Index of 25.00.

### Image analysis

Images were reviewed and analyzed independently using Advantage Workstation (GE Healthcare). Two experienced nuclear medicine physicians (AI and IS, 11 and 6 years of experience interpreting PET scans, respectively) evaluated blindly all PET reconstructions for image quality. Image quality was rated using a 5-point Likert scale (5 excellent diagnostic image quality, 4 good, 3 acceptable, 2 sub-optimal with limited additional clinical information, 1 non-diagnostic) in which a score of 3, 4, or 5 was considered to provide diagnostic value. One of the investigators (IS) identified one representative lesion per patient, and semi-quantitative measurements using maximum standardized uptake values (SUV_max_) were obtained for each reconstruction by drawing a spherical volume-of-interest (VOI) around the selected lesion. Additionally, the liver signal-to-noise ratio (SNR) was obtained for each of the time per bed reconstructions as a quantitative image quality parameter and surrogate of visual image quality. A 3-cm spherical VOI was placed within the right lobe of the liver. Liver SNR defined as the ratio of the voxel mean and the standard deviation of the activity in the VOI [18, 19].

### Statistical analysis

Rating agreement for the image quality assessment between readers was evaluated using kappa statistic. Effects of time per bed on ratings and SNR were estimated by mixed-effects logistic regression with covariates of dose and BMI and random effect of patient. Effects of time per bed on SUV were estimated by mixed-effects GLM log-link regression with covariates of dose and BMI, and random effect of patient. All statistical analyses were done using Stata Release 14.2 (StataCorp LP, College Station, TX, USA). A significance level of 0.05 was used.

## Results

**Fig. 1:**
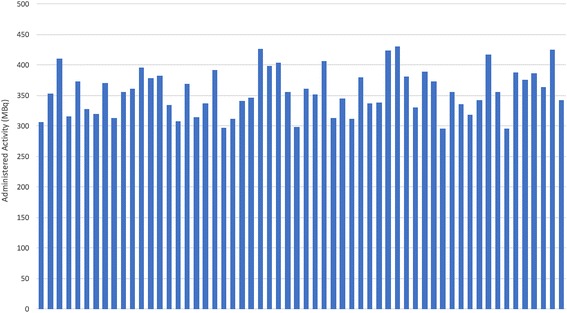
Histogram of administered activity

A total of 58 cancer patients (50 men and 8 women; 27- to 94-year-old, mean ± SD 61.6 ± 15 years) were enrolled in the study; 41 patients had measurable lesions. Patients with all cancer types were included in the study to simulate the actual clinical experience. Relevant demographic and clinical data are showed in Table 1. DMI PET/CT scans were acquired 101.41 ± 17.52 min (range 71–142 min) post-injection of 356.3 ± 37.4 MBq (range 295.3–429.9) of ^18^F-FDG (Fig. 1). This time delay was due to standard PET/CT being acquired first. The wide range was mostly due to different duration of the initial scans, some of them lasting longer (head-to-toes) than others (head-to-mid-thighs) and, in some instances, due to accommodation of specific patient’s needs. Patient’s weight was 81.22 ± 20.28 kg (range 57–143 kg) and body mass index (BMI) was 26.55 ± 5.53 (range 19.79–46.16; median 24.95). The recommended ^18^F-FDG dosage was 370–555 MBq at the time of the DMI installation. Such variations in time from injection to imaging and in administered dosage have been reported at academic medical centers in the USA [20].

**Table 1:** Demographic and clinical data of all study participants

Characteristics	Number of patients
Total number of participants	58
Male	50
Female	8
Average age (± standard deviation) Age range	61.6 years (± 15) 27–94 years
Diagnosis	
Lung cancer	10
Non-Hodgkin lymphoma	9
Head and neck cancer	5
Hodgkin lymphoma	5
Colorectal cancer	4
Melanoma	4
Breast cancer	3
Testicular cancer	2
Renal cell carcinoma	1
Kidney cancer	1
Myocardial sarcoidosis	1
Bladder cancer	1
Endometrial cancer	1
Chronic lymphocytic leukemia	1
Angiosarcoma	1
Hepatocellular carcinoma	1
Esophageal cancer	1
Thyroid cancer	1
Adrenocortical carcinoma	1
Gastric cancer	1
Pulmonary sarcoidosis	1
Adenocarcinoma of unknown primary	1
Lymphoadenopathy, splenomegaly	1
IgA K multiple Myeloma	1
Clinical indication	
Initial treatment strategy	11
Subsequent treatment strategy	47

### Image quality and semi-quantitative assessment

The average scores ± SD of both readers were 2.61 ± 0.83, 3.70 ± 0.92, 4.36 ± 0.82, 4.82 ± 0.39, and 4.91 ± 0.91 for the 30, 60, 90, 120 s/bed and standard acquisition time, respectively.

**Fig. 2:**
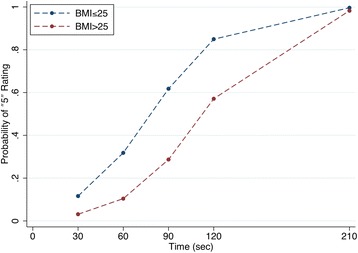
Influence of acquisition time and BMI on image quality

Inter-reader agreement on image quality assessment was good, with a weighted kappa of 0.80 (95% CI 0.72–0.81). Almost half of each reader’s ratings were “5 (Excellent Quality)”; their marginal distributions of ratings were significantly different (marginal homogeneity test *P* < 0.001), and there was a significant tendency for rater 2’s ratings to be higher than rater 1’s when the two disagreed (symmetry test *P* < 0.001) (Tables 2 and 3). Weighted kappa between readers was 0.73 (95% CI 0.66–0.80). There was a significant overall effect of time on ratings (*P* < 0.001) as well as a significant time-by-BMI interaction (*P* < 0.001). There was no significant effect of dose (*P* = 0.052). These findings are summarized in Table 4 and Fig. 2.

**Table 2:** Image quality assessment by the two readers. Rates in the rows are by reader 1 and rates in the columns are by reader 2. Weighted kappa between readers was 0.73 (95% CI 0.66–0.80)

	1	2	3	4	5	Total (%)
1	2	6	2	1	0	11 (3.8%)
2	0	9	13	6	2	30 (10.3%)
3	0	3	27	16	6	52 (17.9%)
4	0	1	9	21	33	64 (22.1%)
5	0	0	1	21	111	133 (45.9%)
Total (%)	2 (0.7%)	19 (6.6%)	52 (17.9%)	65 (22.4%)	152 (52.4%)	290

**Table 3:** Percentage of “5—excellent” ratings in relation to BMI

Time (s)	30	60	90	120	Reference time	Total
BMI ≤ 25	0% 0/60	28% 17/70	75% 45/60	88% 53/60	98% 59/60	58% 174/300
BMI > 25	0% 0/56	7% 4/56	36% 20/56	75% 42/56	82% 46/56	40% 112/280
Total	0% 0/116	18% 21/116	56% 65/116	82% 95/116	91% 105/116	49% 285/580

**Table 4:** Effects of time, BMI, and dose on ratings

Variable	Odds ratio	Standard error	*Z*	*P*	95% confidence interval
Time/30	7.10	1.69	8.25	0.000	4.46–11.31
BMI (vs ≤ 25)	3.38	2.59	1.59	0.112	0.75–15.14
BMI × time/30	0.38	0.10	− 3.85	0.000	0.23–0.62
Dose	1.38	0.23	1.94	0.052	0.99–1.91

**Fig. 3:**
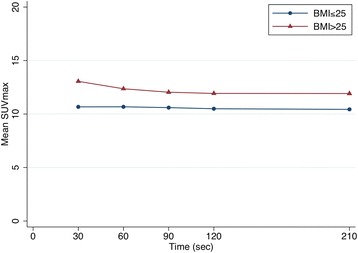
Influence of acquisition time and BMI on SUV max

**Fig. 4:**
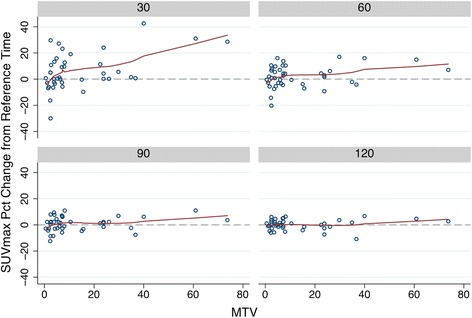
Percentage change from standard time of SUVmax by lesion size. *MTV* mean tumor volume

**Fig. 5:**
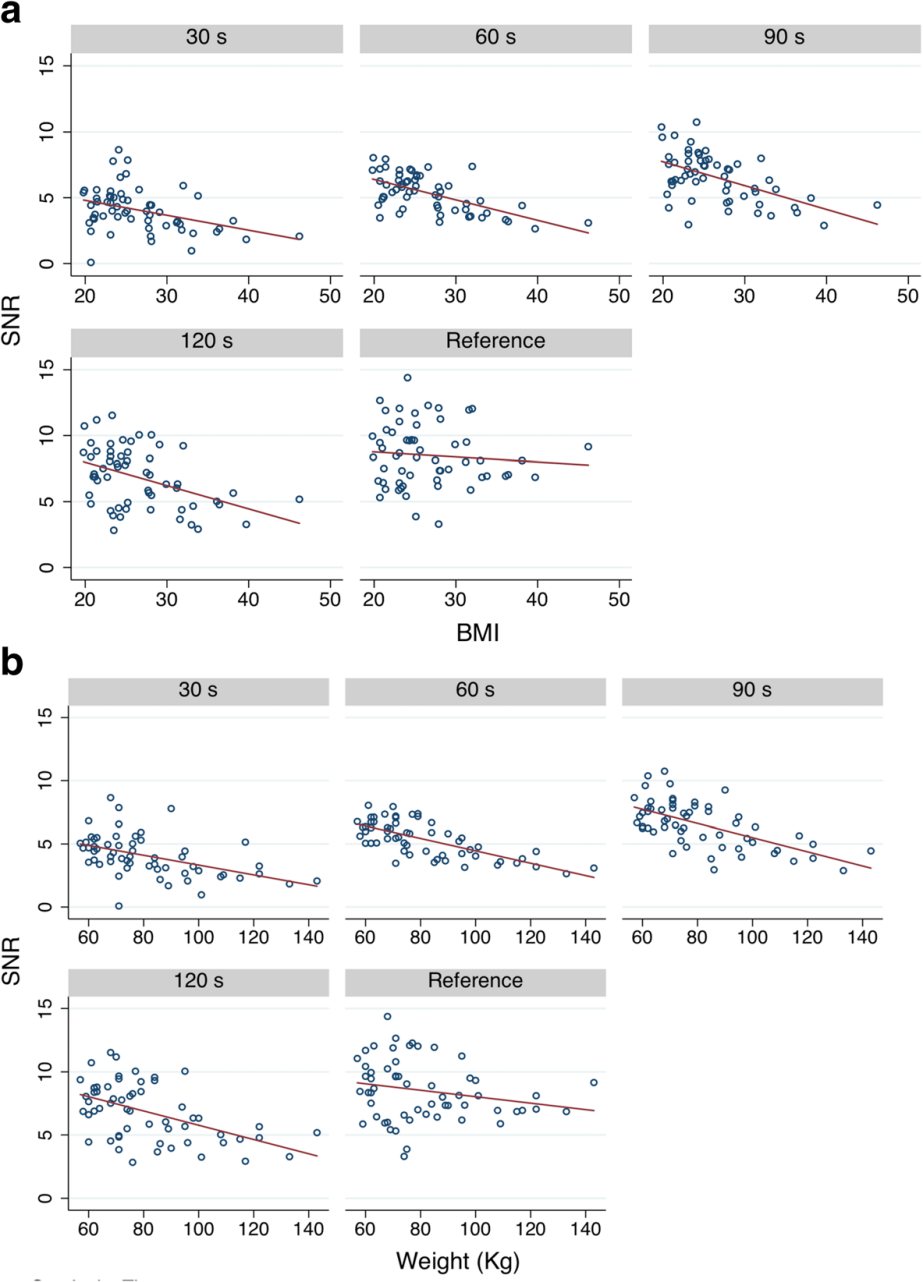
Linear regression analysis correlating liver SNR with BMI (**a**) and body weight in kilogram (**b**)

In the evaluation of the effects of time per bed acquisition on semi-quantitative measurements we found that the only time-point significantly different from the standard time were 30 and 60 s (both with *P* < 0.001). The effects of dose and BMI were not statistically significant (*P* = 0.195 and 0.098, respectively). These are shown in Table 5 and Fig. 3. The percentage change of SUVmax relative to the reference time and to lesion volume is summarized in Table 6 and Fig. 4. The liver SNR obtained with each time per bed reconstruction are summarized in Tables 7 and 8 and Fig. 5a, b. There was a significant positive effect of time on SNR (*P* < 0.001), as well as a significant negative effect of weight (*P* < 0.001), and a possible positive effect of dose (*P* = 0.048). BMI and weight were highly correlated (*r* = 0.90); nevertheless, only weight had a statistically significant effect as predictor of the liver SNR, indicating that it shares more variance with SNR than BMI does.

**Table 5:** Means, SDs, and frequencies of SUV_max_ measurements

	BMI ≤ 25						BMI ≥ 25					
Time (s)	30	60	90	120	Reference time	Total	30	60	90	120	Reference time	Total
Mean	10.67	10.68	10.60	10.49	10.43	10.57	13.06	12.36	12.04	11.92	11.92	12.26
SD	5.4	5.01	4.81	4.66	4.51	4.78	6.40	6.41	6.16	6.05	6.04	6.11
Frequency	20	20	20	20	20	100	21	21	21	21	21	105

**Table 6:** Percentage change of SUV_max_ relative to reference time

Time (s)	Mean	Standard error	95% confidence interval
30	6.01	2.24	1.48–10.53
60	2.08	1.33	− 0.61–4.76
90	0.95	0.88	− 0.81–2.73
120	0.14	0.63	− 1.12–1.14

**Table 7:** Effects of time, BMI, and dose on the liver SNR

Variable	Odds ratio	Standard error	*Z*	*P* > |z|	95% confidence interval
Time/30	0.70	0.04	16.75	0.000	0.62–0.78
Dose	0.29	0.15	1.98	0.048	0.00–0.57
Weight	− 0.05	0.01	− 6.41	0.000	− 0.06–0.03
(Intercept)	4.88	1.49	3.29	0.001	1.97–7.80

**Table 8:** Means, SDs, and frequencies of liver SNR by BMI

	BMI ≤ 25						BMI ≥ 25					
Time (sec)	30	60	90	120	Reference time	Total	30	60	90	120	Reference time	Total
Mean	4.61	5.98	7.28	7.36	8.77	6.80	3.47	4.72	5.75	6.26	8.24	5.69
SD	1.66	1.17	1.77	2.27	2.41	2.35	1.47	1.38	1.59	2.16	2.33	2.41
Frequency	30	30	30	30	30	150	28	28	28	28	28	290

**Fig. 6:**
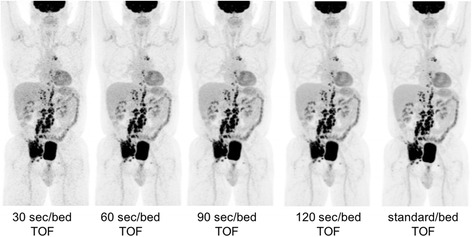
Images acquired 71 min after injection of 423.3 MBq of FDG and reconstructed at various times/bed (BMI 26.6). Reader 1’s scores for each time per bed reconstruction were, respectively, 4, 5, 5, 5, and 5; reader 2’s scores were 3, 4, 5, 5, and 5

**Fig. 7:**
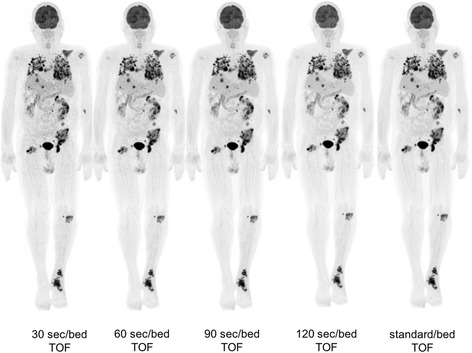
Images acquired 116 min after injection of 330 MBq of FDG and reconstructed at various times/bed (BMI: 21.2). Reader 1’s scores for each time per bed reconstruction were respectively, 2, 4, 5, 5, and 5; reader 2’s scores were 2, 3, 5, 5, and 5

Examples of various reconstructions in two different patients are shown in Figs. 6 and 7.

## Discussion

Among the desired advantages of new PET systems are scanning time and dose reduction and increased image quality which would translate into better patient compliance, better and earlier lesion detection, and ultimately to better patient care and clinical management. Our study suggests that the SiPM technology and TOF reconstruction implemented in the DMI PET/CT scanner allow reduction of PET acquisition time to 90 s/bed, while still producing very good image quality. These may be even better under normal clinical circumstances, as our scans were acquired with a delay due to the standard PET/CT being done first. The high image quality ratings recorded for fast imaging opens other possibilities such as a combination of decreased dosage of the administered radiopharmaceutical and fast imaging, which is now accepted as a compromise in our current use of DMI.

The recent introduction of SiPM detectors has attracted the interest of the scientific community due to the potential advantages of the new technology. These photodetectors are characterized by excellent intrinsic time resolution and high photon detection efficiency. The most attractive advantages derived from the use of SiPM-based PET/CT over standard PET/CT are a reduction in required administered dosage of PET radiopharmaceuticals, higher sensitivity and temporal resolution. Very little is available in the literature on the clinical use of the two technologies combined in PET/MR [15, 16] and PET/CT [21] scanners. A recently published study from our group using the DMI PET/CT system evaluated the performance of the novel scanner, in terms of spatial resolution, sensitivity, noise equivalent count rate (NECR), scatter fraction, count rate accuracy, and image quality with the National Electrical Manufacturers Association (NEMA) NU-2 2012 standards, finding excellent performance [21]. In comparison with other commercially available PET scanners from the same vendor, the DMI PET/CT performed better based on NEMA studies and showed the highest sensitivity, inferior only to the GE SIGNA PET/MR.

An important aspect of our data is that our population included many patients with a BMI > 25. Our study indicates that as expected BMI negatively influences image quality. Due to the higher sensitivity, temporal and spatial resolution of the new technology implemented in the DMI PET/CT, a reduction in acquisition time is possible even in a population with high BMI, while maintaining image quality. It is true that, as expected, image quality and liver SNR declined proportionally with acquisition time reduction, but our data showed that a scanning time of 90 s/bed still allows good image quality with an average score of 4.36 ± 0.82 in a 1 to 5 scale and liver SNR of 6.54 ± 2.27.

Two important aspects that we intend to explore in future studies are the effect of acquisition time reduction on lesion detectability and the possibility of dose reduction. No such analysis was performed in the current study since we chose to focus only on the impact of scanning time duration on image quality and semi-quantitative measurements. One limitation to our study is the delay between injection time and PET/CT acquisition, with an average time between the radiopharmaceutical injection and the beginning of the DMI PET/CT acquisition of 101.41 ± 17.52 min (range 71–142). As already mentioned, this delay was due to the acquisition of a standard of care PET/CT before the DMI PET/CT, which was acquired for research purposes. The delay in acquisition time might influence image quality and SUV measurements, but it is not possible to foresee what kind of impact it might have had on our results. This aspect will be subject of further investigations.

## Conclusions

Despite significant delays from injection to imaging compared to standard clinical operations and even in a population with average BMI > 25, images can be acquired as fast as 90 s/bed using the SiPM-based DMI PET/CT and still result in very good image quality (average score > 4). Fast PET/CT scanning may be significant for pediatric patient (decreasing anesthesia or sedation time) or for sick patients who cannot lay still for prolonged periods of time. In addition, increased patients’ throughput may change workflows in busy clinical practices.
